# Limiting tourniquet use during total knee arthroplasty improves short-term postoperative outcomes in patients with hypertension

**DOI:** 10.3389/fsurg.2025.1535662

**Published:** 2025-02-18

**Authors:** Lei Zhang, Yuhui Ning, Cheng Yang, Tao He

**Affiliations:** Department of Orthopaedic, Qilu Hospital Dezhou Hospital of Shandong University, Dezhou, Shandong, China

**Keywords:** tourniquet, total knee arthroplasty, hypertension, blood loss, rehabilitation, complications

## Abstract

**Introduction:**

Tourniquets are commonly used during total knee arthroplasty (TKA) to reduce intraoperative bleeding. However, information on the optimal tourniquet usage strategy in patients with hypertension are lacking.

**Methods:**

A retrospective analysis of 90 patients with knee osteoarthritis and hypertension who underwent primary TKA was conducted. Patients were divided into three groups: Group A (tourniquet applied before skin incision and released after wound closure), Group B (tourniquet applied before prosthesis placement and released after wound closure), and Group C (tourniquet applied before prosthesis placement and released after bone cement solidification). Outcomes measured included intraoperative and postoperative blood loss, swelling rate, visual analog scale scores, perioperative complications, and Knee Society Scores.

**Results:**

Group A exhibited the lowest intraoperative blood loss volume (239.26 ± 53.83 ml), but this group had significantly higher hidden blood loss, total blood loss, and transfusion volumes than Groups B and C (*P* < 0.05). The swelling rate and visual analog scores on postoperative day 3 were also significantly higher in Group A than in Groups B and C, as was the incidence of complications, including anemia, deep vein thrombosis, and anterior knee pain. Knee Society Scores at 4 weeks postoperatively were significantly lower in Group A than in Groups B and C.

**Discussion:**

Tourniquet application before prosthesis placement and release after wound closure or bone cement solidification were associated with better short-term outcomes, reduced complications, and improved rehabilitation compared to tourniquet use during the entire procedure in patients with hypertension undergoing TKA.

## 1 Introduction

The prevalence of knee osteoarthritis is rapidly increasing owing to the aging population, imposing a substantial social and economic burden. End-stage knee osteoarthritis is characterized by debilitating pain, joint deformity, and impaired mobility (1, 2). The most effective intervention for end-stage knee osteoarthritis is total knee arthroplasty (TKA), which significantly enhances patient quality of life (3, 4). However, TKA can lead to considerable blood loss due to extensive soft tissue and bone trauma, resulting in complications such as surgical site infections, anemia, and suboptimal functional recovery. These complications can impede early rehabilitation and increase the risk of thrombotic events (5–7). Consequently, the development of strategies to minimize intraoperative blood loss has become a critical focus in orthopedic research. Hypertension, a common chronic condition characterized by persistently elevated blood pressure (≥140/90 mmHg), is a significant global health issue associated with increased cardiovascular risk and systemic complications (8, 9). In surgical patients, particularly those undergoing TKA, hypertension further exacerbates the risk of perioperative bleeding and complications (10). Understanding the interplay between hypertension and surgical outcomes is therefore essential for optimizing perioperative management strategies in this subgroup.

Tourniquets are commonly used in TKA to reduce intraoperative bleeding, provide a dry and clear osteotomy field, minimize blood contamination of bone cement, enhance surgical precision, and facilitate implant fixation (11, 12). However, tourniquet application is also associated with several disadvantages, including hidden blood loss, thigh pain, nerve palsy, ischemia, soft tissue injury, thromboembolic complications, impaired wound healing, and patellar maltracking. Additionally, tourniquet use may delay patient recovery by reducing quadriceps femoris muscle strength, decreasing knee range of motion, and exacerbating postoperative pain (13–15).

Numerous randomized controlled trials and meta-analyses have assessed the use of tourniquets during TKA, although the optimal tourniquet release time and whether its use is beneficial throughout the procedure remain controversial (16–18). However, most of these studies have involved general patient populations, without specifically examining patient subgroups that are more susceptible to blood loss. Hypertension increases the risk of intraoperative bleeding in patients undergoing TKA, and these patients are more likely to experience cardiovascular and perioperative complications. Research investigating optimal tourniquet application strategies in patients with hypertension undergoing TKA is lacking. The aim of this study was therefore to compare different tourniquet application methods during TKA in patients with hypertension and identify the most effective and safest technique for tourniquet use in this population.

## 2 Method

### 2.1 Selection criteria

Prior to the commencement of this study, ethical approval was obtained from the Institutional Review Board of Qilu Hospital, Dezhou Hospital of Shandong University. The authors assume full responsibility for ensuring the integrity and accuracy of the study data. The inclusion criteria were as follows: (1) complete clinical and follow-up data, (2) Kellgren–Lawrence radiographic classification grade III–IV, (3) diagnosis of hypertension, and (4) primary TKA indicated after failed conservative treatment. The exclusion criteria were as follows: (1) bilateral TKA or revision surgery, (2) knee varus or valgus deformities exceeding 15°, (3) diagnosis of rheumatoid arthritis, (4) TKA not performed, and (5) incomplete follow-up data.

### 2.2 Patients

A total of 90 patients with knee osteoarthritis and hypertension who underwent primary TKA at Qilu Hospital, Dezhou Hospital of Shandong University, between July 2021 and December 2022 met the eligibility criteria and were included in the study. The patients were divided into three groups: A, B, and C. Group A included 15 males and 15 females with a mean age of 68.81 ± 8.17 years, Group B included 14 males and 16 females with a mean age of 69.63 ± 8.75 years, and Group C included 13 males and 17 females with a mean age of 67.86 ± 8.26 years. There were no significant differences among the three groups in terms of baseline characteristics, including age, sex, affected side, disease duration, body mass index, preoperative Knee Society Score (KSS), or hemoglobin levels (*P* > 0.05). Detailed baseline data are presented in Table 1.

**Table 1:** Baseline patient characteristics among the three groups.

Parameter	Age (years)	Sex (male/female)	Disease duration (months)	BMI (kg/m^2^)	Affected side (left/right)	KSS score	Preoperative Hb (g/L)
Group A	68.81 ± 8.17	15/15	28.23 ± 5.62	31.59 ± 4.69	13/17	43.59 ± 4.69	100.23 ± 13.28
Group B	69.63 ± 8.75	14/16	29.26 ± 7.58	32.62 ± 5.37	15/15	39.26 ± 7.58	108.31 ± 12.54
Group C	67.86 ± 8.26	13/17	31.82 ± 6.65	30.56 ± 4.71	13/17	41.82 ± 5.36	105.95 ± 12.93
F/*χ*^2^ *value	1.331	0.692*	1.692	2.031	0.958*	2.528	1.853
*P* value	0.826	0.326	0.537	0.269	0.276	0.318	0.362

Data are presented as mean ± standard deviation.

* χ^2^ values are presented for categorical variables (sex and affected side). BMI, body mass index; Hb, hemoglobin; KSS, knee society score.

### 2.3 Preoperative intervention

All patients underwent preoperative management to ensure optimal blood pressure control. All patients were instructed to continue their routine antihypertensive medications at home until the day before surgery. Patients with grade 1 or 2 hypertension were maintained on their usual antihypertensive regimen, with no additional preoperative interventions. For patients with grade 3 hypertension, active efforts were made to stabilize and normalize blood pressure to within safe limits for surgery; when necessary, this involved multidisciplinary consultations with relevant specialists to design tailored treatment regimens. TKA was only performed after achieving satisfactory blood pressure control, to minimize the risk of intraoperative and postoperative complications.

### 2.4 Surgical procedure

To minimize bias, all surgeries were performed by the same experienced surgeon using the same cemented knee prosthesis (NexGen Complete Knee Solution; Zimmer Biomet, Warsaw, IN, USA). In Group A, the tourniquet was applied before skin incision and released after wound closure. In Group B, the tourniquet was applied before the placement of the knee prosthesis and released after wound closure. In Group C, the tourniquet was applied before prosthesis placement and released after the bone cement had fully solidified. All patients were placed in the supine position, and a conventional anterior medial incision was used to expose the knee joint. The medial parapatellar approach was employed to incise the joint capsule and expose the joint cavity. This facilitated the removal of the synovium, part of the fat pad, the meniscus, the anterior cruciate ligament, and osteophytes. Subsequently, osteotomies of the tibia, femur, and patella were performed. A trial prosthesis was used to achieve soft tissue balance, eversion stability, and restoration of the mechanical axis of the lower limb before implanting the actual knee prosthesis. Finally, the incision was meticulously closed in layers, including the muscles, fascia, and soft tissue, and a sterile dressing was applied (19).

### 2.5 Postoperative intervention

All patients were provided with intravenous patient-controlled analgesia for pain management and received routine postoperative anti-inflammatory and anticoagulation therapy. Additionally, all patients were prescribed oral etoricoxib 60 mg twice daily for pain relief following surgery. Blood transfusions were considered for patients with hemoglobin levels below 70 g/L. Patients with hypoproteinemia (albumin levels <30 g/L) were administered with intravenous albumin (CSL Behring, King of Prussia, PA, USA). Cane- or walker-assisted functional mobility exercises were initiated on the day of surgery. From the second postoperative day until discharge, patients participated in physiotherapist-led rehabilitation exercises. At discharge, patients were provided with a structured home-based exercise program to maintain functional recovery.

### 2.6 Observation indicators

Operation time, intraoperative blood loss, and postoperative complications were recorded for each patient. Hidden blood loss, total blood loss, blood transfusion volume, swelling rate, and visual analog scale pain scores were assessed on the third postoperative day. Total blood loss was estimated using the Gross formula (20, 21). The KSS was calculated at 4 weeks and 12 months postoperatively.

### 2.7 Statistical analysis

All data were analyzed using SPSS software version 25.0 (IBM Corporation, Armonk, NY, USA). Continuous variables are expressed as mean ± standard deviation and categorical variables are presented as frequencies. The Shapiro–Wilk test was first used to assess the normality of continuous data. Normally distributed data were compared using independent sample t-tests; non-normally distributed data were compared using the Mann–Whitney *U* test. Categorical data were analyzed using the chi-square test. *P* < 0.05 was considered statistically significant.

## 3 Results

### 3.1 Blood loss

**Figure 1:**
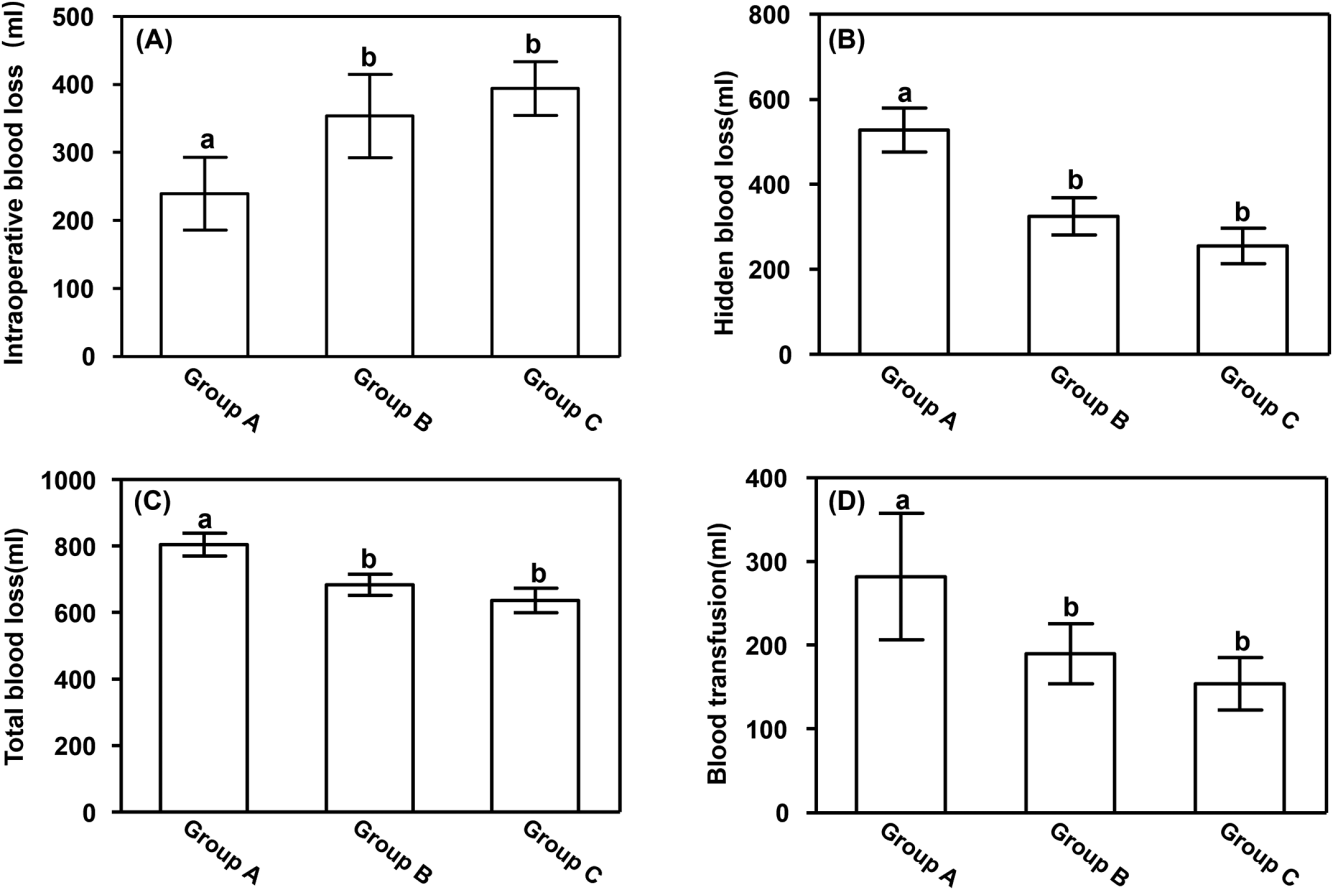
Blood loss. **(A)** Intraoperative blood loss, **(B)** hidden blood loss, **(C)** total blood loss, and **(D)** blood transfusion volume. Data are presented as mean ± standard deviation. ^a^*P* < 0.05 vs. groups B and C; ^b^*P* < 0.05 vs. group A.

Intraoperative blood loss was significantly lower in Group A (239.26 ± 53.83 ml) than in Groups B (353.72 ± 61.36 ml) and C (394.28 ± 39.61 ml) (*P* < 0.05). However, hidden blood loss, total blood loss, and blood transfusion volume were all significantly higher in Group A than in Groups B and C (*P* < 0.05), as shown in Figure 1.

### 3.2 Perioperative parameters

**Figure 2:**
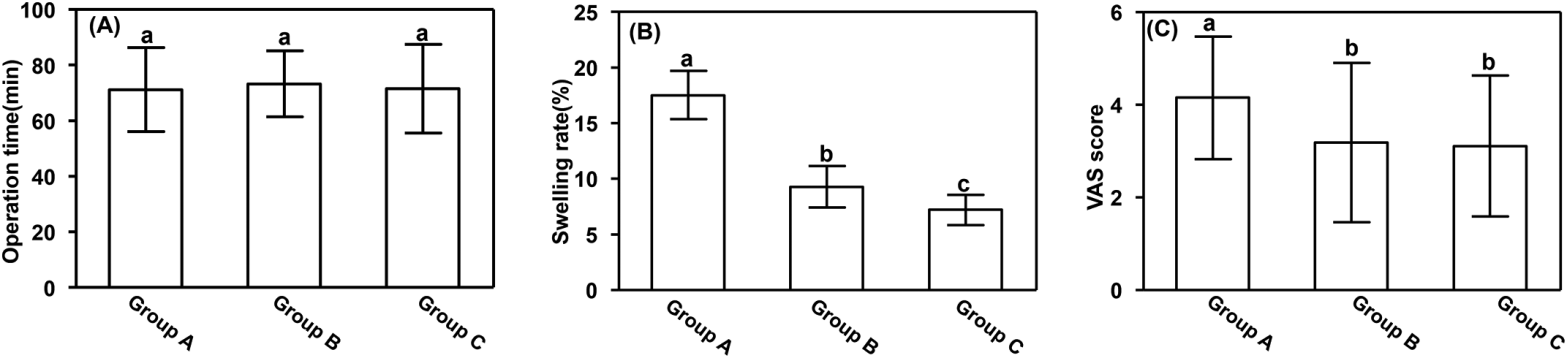
Perioperative parameters. **(A)** Operation time, **(B)** swelling rate, and **(C)** VAS score. Data are presented as mean ± standard deviation. ^a^*P* < 0.05 vs. groups B and C; ^b^*P* < 0.05 vs. group A. VAS, visual analog scale.

No significant differences in operation time were observed among the groups. However, the swelling rate and visual analog scale scores on the third postoperative day were significantly higher in Group A (17.52% ± 2.17% and 4.15 ± 0.38, respectively) than in Groups B (9.28% ± 1.85% and 3.18 ± 1.72, respectively) and C (7.21% ± 1.37% and 3.11 ± 1.52, respectively) (*P* < 0.05). Additionally, the swelling rate was significantly higher in Group B than in Group C (*P* < 0.05). These results are presented in Figure 2.

### 3.3 Perioperative complications

All patients demonstrated good postoperative recovery and satisfactory wound healing. In Group A, one patient developed deep vein thrombosis, three experienced anemia, and one reported anterior knee pain, a complication rate of 17.8%. In Group B, one patient developed venous thrombosis, a complication rate of 3.3%. No complications were reported in Group C. No cases of acute myocardial infarction, cerebral infarction, or pulmonary embolism were observed in any of the three groups. The complication rate in Group A was significantly higher than those in Groups B and C (*P* < 0.05).

### 3.4 Follow-up

**Figure 3:**
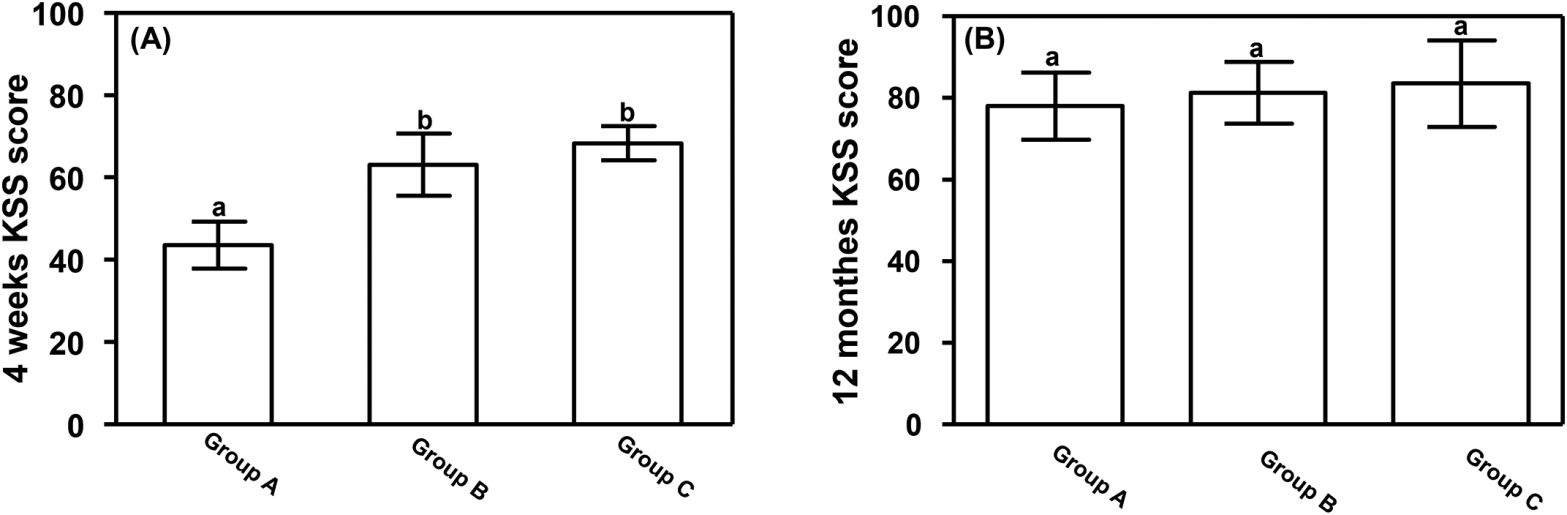
Postoperative KSS. **(A)** Four weeks and **(B)** 12 months after total knee arthroplasty. Data are presented as mean ± standard deviation. ^a^*P* < 0.05 vs. groups B and C; ^b^*P* < 0.05 vs. group A. KSS, knee society score.

All patients were followed up for 12 months. Four weeks after TKA, the KSS was significantly lower in Group A (43.56 ± 5.71) than in Groups B (63.15 ± 7.56) and C (68.29 ± 4.19) (*P* < 0.05). However, 12 months after TKA there were no significant differences in KSS among the three groups (*P* > 0.05), as show in Figure 3.

## 4 Discussion

Hypertension significantly impairs the structural integrity of the vascular wall. Patients with hypertension typically experience elevated blood pressure, which contributes to increased bleeding during and after surgery (22, 23). Numerous studies have demonstrated that patients with knee osteoarthritis and hypertension undergoing TKA experience significantly greater intraoperative and postoperative blood loss compared to patients without hypertension (24, 25). As a result, patients with hypertension, particularly when accompanied by other diseases, often require greater volumes of blood transfusions during surgery. Park et al. identified hypertension as the second most common preoperative comorbidity after anemia in patients undergoing arthroplasty, highlighting the importance of addressing this issue (5). During TKA, patients with hypertension experience increased bleeding during skin incision and exposure, likely due to fluctuations in blood pressure during the operation. Even when blood pressure is controlled within an acceptable range, systolic pressure may still spike, increasing blood loss (24). Additionally, hemostasis techniques, such as electrocoagulation or gauze compression, are often less effective in patients with hypertension than in those without hypertension, further contributing to intraoperative blood loss (26). Consequently, tourniquet use during TKA is of critical importance in patients with hypertension to mitigate perioperative blood loss (27–29). In this study, patients were divided into three groups, each subjected to different tourniquet application strategies to evaluate their effects. The results showed that intraoperative blood loss in Groups A and B was significantly lower compared to Group C. However, both Groups A and B demonstrated a significant increase in hidden blood loss, leading to a higher total blood loss compared to Group C. These findings suggest that although tourniquet use has certain advantages in reducing intraoperative bleeding, it may increase the risk of hidden blood loss, potentially impacting postoperative recovery in hypertensive patients. This underscores the importance of achieving a balance between intraoperative and postoperative blood loss control when applying intermittent tourniquet strategies in hypertensive patients undergoing knee arthroplasty. These findings provide valuable insights for developing individualized tourniquet application protocols to optimize clinical outcomes in this patient population.

Prolonged tourniquet use has been associated with higher postoperative pain scores and delayed functional recovery following total knee arthroplasty (TKA). In this study, patients with continuous tourniquet application experienced more early postoperative pain and swelling compared to those with intermittent use. The reduced pain observed with intermittent tourniquet use is likely due to several factors: prolonged application causes localized ischemia and reperfusion injury, which can trigger cell damage and inflammation, exacerbating pain. Reducing the duration of tourniquet use minimizes ischemia and reperfusion injury, alleviating pain. Additionally, prolonged use can compress or ischemically affect surrounding nerves, leading to neuropathic pain, which may be reduced by shortening the tourniquet application. Tourniquet use also induces tissue hypoxia and inflammatory responses, including the release of pro-inflammatory cytokines, which can worsen postoperative pain. Limiting the duration of use may decrease these inflammatory responses and reduce pain. Furthermore, the ischemic pressure on muscles and soft tissues can cause muscle damage and significant pain. Reducing tourniquet use can lessen this pressure and decrease muscle-related pain (30). Prolonged tourniquet use may also inhibit the restoration of local blood flow, prolonging pain. By reducing its duration, blood flow recovery is expedited, helping to alleviate pain. However, these effects did not significantly impact long-term outcomes (31). Therefore, minimizing tourniquet use during TKA, particularly in hypertensive patients, is recommended. A study by Olivecrona et al., which included 577 patients undergoing primary TKA and 46 undergoing revision TKA, demonstrated a significant increase in complication risk with every additional 10 minutes of tourniquet application (32). This finding aligns with the conclusions of the present study, further confirming the importance of personalized tourniquet use in optimizing surgical outcomes during joint replacement procedures.

Tourniquet use requires careful consideration, as it is associated with a substantially increased risk of deep vein thrombosis and intermuscular vein thrombosis. These findings underscore the importance of balancing the hemostatic benefits of tourniquet use with its potential risks, particularly in patients at a higher risk of thromboembolic complications (24). Complications became markedly more frequent when tourniquet duration exceeded 100 min (32). Ozkunt et al. conducted a prospective randomized study of 69 patients using three distinct tourniquet application methods during TKA, and reported that prolonged use did not enhance bone cement infiltration but was associated with an increased visual analog scale score and a decreased KSS (33). Similarly, a randomized double-blind controlled trial by Wang et al. demonstrated that limiting tourniquet application to the implant placement phase did not increase transfusion rates or surgical time compared with continuous tourniquet use, but did reduce pain on the first postoperative day, allow the earlier initiation of straight leg-raising exercises, and decrease the number of minor complications experienced (17). Cai et al. further reported that periodic tourniquet application effectively reduced postoperative blood loss and total blood loss, mitigated limb swelling, and facilitated early rehabilitation (13). In this study, patients in Group A had a continuous use of the tourniquet throughout the entire TKA procedure, whereas patients in Groups B and C used the tourniquet intermittently, with Group C having a shorter tourniquet application time compared to Group B. In Group A, one patient developed deep vein thrombosis, three experienced anemia, and one reported anterior knee pain, resulting in a complication rate of 17.8%. In Group B, one patient developed venous thrombosis, leading to a complication rate of 3.3%. No complications were reported in Group C. The results of the study confirm that reducing the duration of tourniquet use significantly lowers the incidence of complications. Furthermore, tourniquets applied during osteotomy, prosthesis implantation, and from osteotomy to wound closure significantly reduced postoperative hemorrhage and swelling rates compared with continuous tourniquet use. This may be due to a decrease in vascular wall injury, muscle ischemia-reperfusion injury, and rhabdomyolysis caused by prolonged tourniquet application. These effects exacerbate postoperative bleeding and severe limb swelling, ultimately impairing early functional recovery.

Patients with hypertension undergoing TKA are at an increased risk of tourniquet-associated adverse effects owing to their more fragile vascular walls and reduced regulatory function, which render them more susceptible to sudden cardiovascular events under conditions of stress. Tourniquet application can stimulate the sympathetic nervous system, causing significant hemodynamic fluctuations that heighten the risk of myocardial ischemia or acute myocardial infarction during surgery (34, 35). Anesthesiologists play a critical role in mitigating these risks by effectively controlling the stress response and maintaining hemodynamic stability through the use of drugs that inhibit sympathetic activity. In addition to cardiovascular risks, tourniquets can impair pulmonary function. Patients with hypertension often experience vascular endothelial dysfunction, leading to an imbalance in endothelin and nitric oxide levels, which may negatively affect lung function (36).

In the present study, the incidence of postoperative anemia, deep vein thrombosis, and intermuscular venous thrombosis was significantly higher in patients who underwent prolonged tourniquet application than in those for whom tourniquet use was limited. When using tourniquets in patients with hypertension, careful consideration of the duration and timing of their application is essential, and close intraoperative monitoring by anesthesiologists is crucial to prevent adverse reactions. With the ongoing development of robotic-assisted technology, increasing evidence suggests that robotic systems can provide higher precision in intraoperative alignment, avoiding the use of intramedullary alignment rods, which may help reduce both intraoperative and postoperative bleeding, thus improving recovery outcomes. The use of robotics can ensure accurate limb alignment, minimizing damage to blood vessels and soft tissues, which in turn reduces the risk of bleeding and accelerates rehabilitation. Studies have shown that robotic-assisted surgery improves alignment accuracy, not only enhancing functional outcomes but also decreasing excessive blood loss and the need for postoperative interventions (37–40). Given the higher bleeding risks in hypertensive patients, these precise technologies could have a more positive impact on their postoperative recovery, and thus, further studies should evaluate the potential of robotic assistance in this population.

Additionally, although tourniquet use is widely accepted in surgeries, postoperative pain remains a significant concern. The use of a tourniquet is associated with increased postoperative pain, likely due to ischemic tissue damage and nerve stimulation. To alleviate postoperative pain and promote recovery, certain strategies can be considered. One approach is the use of local anesthesia techniques, such as periarticular injections or continuous nerve blocks, which have been shown to reduce pain intensity and improve early mobilization (41, 42). Furthermore, reducing the duration of tourniquet use, employing controlled hypotension, or utilizing modified anesthetic techniques may also help mitigate the severity of postoperative pain. These methods could improve the overall recovery experience for TKA patients.

Despite the valuable insights provided by this study, several limitations should be acknowledged. First, the small sample size may limit the generalizability of the findings. A larger cohort would enhance data robustness and enable subgroup analyses. Second, using hemoglobin (Hgb) levels to estimate blood loss, while common, has limitations in accurately reflecting both intraoperative and hidden blood loss. More precise methods, such as radiolabeled red blood cell studies, were not feasible in this retrospective study. Third, this study focused only on hypertensive patients, and the findings may not apply to normotensive individuals or those with other comorbidities. Fourth, while the study demonstrates that limited tourniquet use reduces postoperative pain, the underlying pathophysiological mechanisms remain unexplored. Further research is needed to clarify these mechanisms. Additionally, while endothelin and nitric oxide's effects on pulmonary function were mentioned, their correlation with respiratory outcomes was not fully explored. A more comprehensive investigation, supported by quantitative data, would strengthen the manuscript. Finally, as a single-center study, the results may be influenced by institutional protocols and surgeon-specific techniques, which could limit their external validity. Future studies should address these limitations by including larger, multicenter cohorts, using more accurate blood loss estimation techniques, and exploring the biological mechanisms of tourniquet use. Long-term studies are also needed to evaluate the impact of different tourniquet strategies on knee function recovery in hypertensive patients undergoing TKA.

In conclusion, the results of the present study indicate that applying the tourniquet before placing the knee prosthesis and loosening it after wound closure and bandage application can effectively reduce intraoperative blood loss, improve short-term postoperative functional outcomes, and reduce the complication rate. However, the long-term clinical effects require further investigation. Given the increased risk of cardiovascular, pulmonary, and thrombotic complications, patients with hypertension require a cautious and individualized approach to tourniquet use during TKA.

## Data Availability

The raw data supporting the conclusions of this article will be made available by the authors, without undue reservation.
